# Patient-reported outcome (PRO) measurements in chronic and malignant diseases: ten years’ experience with PRO-algorithm-based patient-clinician interaction (telePRO) in AmbuFlex

**DOI:** 10.1007/s11136-022-03322-9

**Published:** 2023-01-13

**Authors:** Niels Henrik I. Hjollund, Louise Pape Larsen, Annette Ladefoged de Thurah, Birgith Engelst Grove, Halla Skuladottir, Hanne Linnet, Rasmus Blechingberg Friis, Søren Paaske Johnsen, Ole May, Annesofie Lunde Jensen, Troels Krarup Hansen, Gry Assam Taarnhøj, Lærke Kjær Tolstrup, Helle Pappot, Per Ivarsen, Liv Dørflinger, Anne Jessen, Nanna Toxvig Sørensen, Liv Marit Valen Schougaard, The AmbuFlex team

**Affiliations:** 1 AmbuFlex - Center for Patient-Reported Outcomes, Central Denmark Region, Gødstrup Hospital, Herning, Denmark; 2 Department of Clinical Epidemiology, Aarhus University Hospital, Aarhus, Denmark; 3 Department of Clinical Medicine, Aarhus University, Aarhus, Denmark; 4 Department of Rheumatology, Aarhus University Hospital, Aarhus, Denmark; 5 Department of Oncology, Gødstrup Hospital, Herning, Denmark; 6 Danish Center for Clinical Health Services Research, Department of Clinical Medicine, Aalborg University, Aalborg, Denmark; 7 Department of Medicine, Gødstrup Hospital, Herning, Denmark; 8 Steno Diabetes Center Aarhus, Aarhus, Denmark; 9 Department of Oncology, University of Copenhagen, Rigshospitalet, Copenhagen, Denmark; 10 Department of Renal Medicine, Aarhus University Hospital, Aarhus, Denmark; 11 The Danish Cancer Society, Copenhagen, Denmark

**Keywords:** Algorithm, Chronic disease, Decision support systems, Malignant diseases, Outpatient follow-up, Patient-reported outcome measures, Questionnaires

## Abstract

**Background:**

Patient-reported Outcome (PRO) measures may be used as the basis for out-patient follow-up instead of fixed appointments. The patients attend follow-up from home by filling in questionnaires developed for that specific aim and patient group (telePRO). The questionnaires are handled in real time by a specific algorithm, which assigns an outcome color reflecting clinical need. The specific questionnaires and algorithms (named solutions) are constructed in a consensus process with clinicians. We aimed to describe AmbuFlex’ telePRO solutions and the algorithm outcomes and variation between patient groups, and to discuss possible applications and challenges.

**Methods:**

TelePRO solutions with more than 100 processed questionnaires were included in the analysis. Data were retrieved together with data from national registers. Characteristics of patients, questionnaires and outcomes were tabulated for each solution. Graphs were constructed depicting the overall and within-patient distribution of algorithm outcomes for each solution.

**Results:**

From 2011 to 2021, 29 specific telePRO solutions were implemented within 24 different ICD-10 groups. A total of 42,015 patients were referred and answered 171,268 questionnaires. An existing applicable instrument with cut-off values was available for four solutions, whereas items were selected or developed ad hoc for the other solutions. Mean age ranged from 10.7 (Pain in children) to 73.3 years (chronic kidney disease). Mortality among referred patients varied between 0 (obesity, asthma, endometriosis and pain in children) and 528 per 1000 patient years (Lung cancer). There was substantial variation in algorithm outcome across patient groups while different solutions within the same patient group varied little.

**Discussion:**

TelePRO can be applied in diseases where PRO can reflect clinical status and needs. Questionnaires and algorithms should be adapted for the specific patient groups and clinical aims. When PRO is used as replacement for clinical contact, special carefulness should be observed with respect to patient safety.

**Supplementary Information:**

The online version contains supplementary material available at 10.1007/s11136-022-03322-9.

## Background

The term Patient-reported Outcome (PRO) was coined by the US Federal Drug Agency to standardize the use of such data to support labeling claims in medical product development [1]. Interest in using PRO data, also at the individual patient level, is growing [2–4]. PRO data can be used during the consultation as a tool to support communication, and research has shown benefits in terms of process measures such as improved patient-clinician communication and better detection of problems [5–8]. However, when patients fill in PRO data at a distance (telePRO), e.g. at home, PRO data can be processed before the consultation, providing information that may enhance flexibility in care and more efficient use of health care services without compromising quality of care [3, 9–11]. In some cases, quality of care may even be improved as measured by quality of life and survival rates [12–14].

### Development of telePRO

Patients with chronic and malignant diseases have variable need of clinical attention. If they attend standardized out-patient follow-up, they may not need attention on the day of a fixed appointment, resulting in waste of transport to hospital, patient time, and clinician time. In telePRO, PRO constitutes the basis for the contact and fixed appointments are replaced with fixed questionnaires as the basis for follow-up. The questionnaire is filled in at home, and an appointment is made only if the questionnaire indicates a need or a patient wish of a consultation. The decision to be referred to AmbuFlex as well as to revert to standard follow-up is taken by the clinician together with the patient. TelePRO may also be used for other purposes, enhancing flexibility (see below). Based on an existing system for PRO data collection for group level use [15, 16], we developed the telePRO system AmbuFlex [17]. The aim was four-fold: first, to improve quality of care by flagging important symptoms and produce better documentation for the patient record; second, to promote patient-centered care with focus on patients’ needs and knowledge about own disease; third, to optimize the use of resources in the healthcare system, and finally, to use the PRO data in research and hospital quality assurance [17]. AmbuFlex, Center for Patient-reported Outcomes, is a part of the public hospital organization in Region Central Denmark, where we since 2011 have developed and implemented telePRO in chronic and malignant diseases, also in other parts of Denmark. The development is a teamwork with 27 employees including with a health professional background, software developers, quality assurance specialists, and health researchers. Apart from algorithm-based telePRO, AmbuFlex has also implemented clinical PRO in the traditional way, where PRO is used solely to promote communication and consultation quality.

### Use of telePRO

**Fig. 1:**
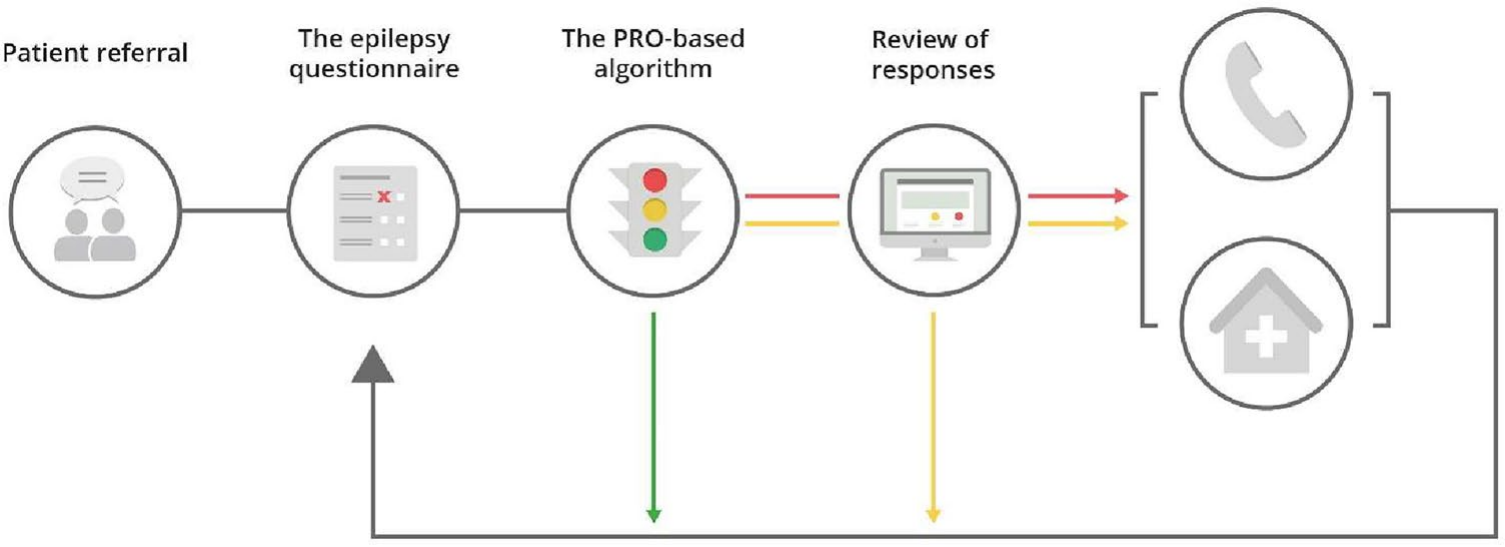
Patient pathways in PRO-algorithm-based follow-up. Example: AmbuFlex/epilepsy. Patients are individually referred by the patient’s clinician. Patients complete a telePRO questionnaire developed for that specific patient group and aim at pre-defined individual intervals, e.g., 3 months. The system prompts patients to fill in the PRO through “e-Boks” (secure national e-mail platform). The epilepsy telePRO includes 47 items covering number of seizures, medicine adherence, symptoms, general health, and psychosocial function measured using the WHO-5, items from the SF-36, SCL-92 and ad hoc developed items. An item covers the patient’s wish of contact to ensure that patients always can get an appointment. As part of development, an expert group has marked the response categories in the telePRO with a green, yellow, or red color based on a flag approach. Red flag: need of clinical attention (e.g., planning pregnancy, seizure impairments, suicidal thoughts, or if the patient wishes contact). A green flag indicates no need of clinical attention, a yellow flag possible need of attention, and a red flag need of attention. “All-green” outcomes are managed automatically by the AmbuFlex system and a new telePRO is sent to the patient at the pre-defined interval, while red and yellow algorithm outcomes are reviewed by a clinician (Fig. 2). (Color figure online)

**Fig. 2:**
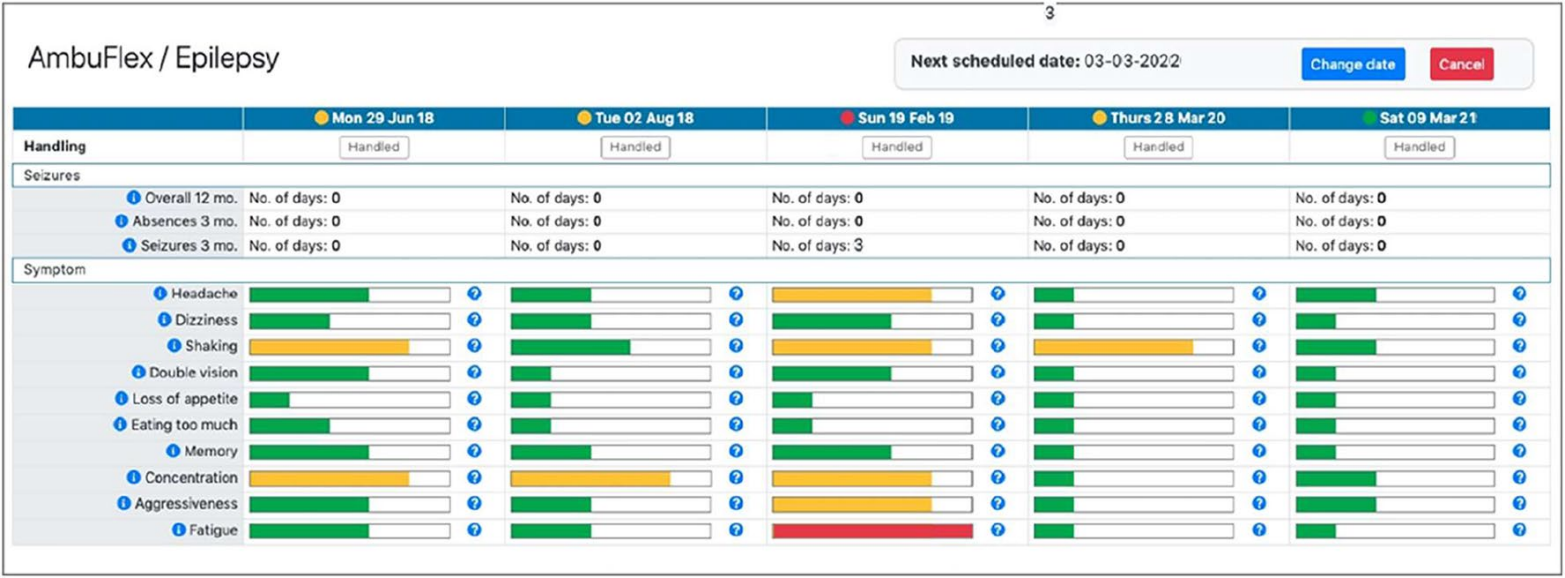
Screenshot of the clinician’s PRO overview. Example: AmbuFlex/epilepsy. The telePRO responses are presented in a graphic overview inside the electronic health record (EHR) system. All red and yellow algorithms outcomes are shown to the clinicians on an alert list. For red outcomes, the clinicians contact the patient either by telephone or by an in-clinic appointment. For yellow outcomes, the clinicians evaluate the PRO data together with other available data and contacts the patient if necessary. (Color figure online)

Algorithm-based telePRO consist of three elements: the PRO data, the PRO-based algorithm, and the presentation of the PRO measures in a graphical overview [17]. The technology for the elements is generic, but configurable for each solution (each specific patient group and clinical aim), e.g., screening for symptom deterioration and need of type of contact and as a treatment decision tool. In a solution with the main purpose to screen for the patients’ need of contact, a green, yellow, or red algorithm outcome color is used based on a “red flag” approach. A green outcome reflects no actual need of clinical attention. However, the patients are allowed to overrule the PRO-based algorithm by indicating a wish for contact. A questionnaire has a red outcome if just one item in the algorithm is flagged red, while a green outcome is applied if all flags are green. All other questionnaires have a yellow algorithm outcome. Since the algorithms are solution-specific, the meaning and consequence of the outcome colors differ between solutions. In some solutions, green outcomes are handled automatically by the AmbuFlex software, while yellow and red outcomes are reviewed and evaluated by a clinician. The principle of AmbuFlex is further explained in Figs. 1 and 2. The development of the solution-specific questionnaires and algorithms is described elsewhere [17].

### Aim

The aim of this paper was (1) to provide an overview of all AmbuFlex’s specific telePRO solutions, (2) to describe the algorithm outcomes and variation in outcomes, (3) to discuss similarities and differences between patient groups in terms of demographic characteristics and algorithm outcomes, and (4) to highlight possibilities and challenges in the use of telePRO.

## Material and methods

### Selection of solutions

Included in the analysis were AmbuFlex solutions using algorithms developed for research or routine use if more than 100 processed questionnaires were available. Solutions in identical patient groups using similar questionnaires and algorithms were merged before analysis.

### Data collection

Questionnaire data and the results of the algorithms were retrieved from the internal database together with information on the patient’s sex, age, and vital status and was last updated January 15, 2022. Information on vital status is automatically retrieved online by the AmbuFlex system from the Danish civil registration system [18]. Mortality of referred patients was calculated for each solution with person-years measured from the date of response to the patient’s first questionnaire to the date of death or the last vitality status update. Total observation time in AmbuFlex is the sum of patient’s individual time span between the date of first and last answered questionnaire per solution. Information on algorithm outcome is recorded in the AmbuFlex system for each questionnaire with the outcome colors green, yellow, or red.

### Data analysis

Descriptive tables were constructed using AmbuFlex’s own software [15]. Algorithm outcomes were anonymized and transferred for further analysis in the R statistical software package [19]. The ranking of the three algorithm outcome colors is the same for all solutions (red is more severe than yellow, which is more severe than green). In most solutions, the difference in consequences between a yellow and a red algorithm outcome is smaller than the difference between a green and a yellow outcome. In some solutions only two colors were applied (green/red or yellow/red). To allow comparison across solutions, severity grade values of 0, 2, and 3 were assigned to green, yellow, and red outcomes and used to rank the questionnaires from each patient (Table 1). Each questionnaire can have one of three outcome colors, and therefore a patient with at least three answered questionnaires may have one of seven combinations of algorithm outcomes (severity group). Graphs were constructed for each solution depicting the frequency and variation in algorithm outcomes. Before plotting, patients were sorted by severity group. The total area of each color represents the overall proportion of that algorithm outcome, while the within-group variation is represented for each severity group. Components of variation in algorithm outcome severity score (within- and between-patient) were calculated for solutions with more than one answer from each patient. The anovaVCR function in the R VCR package was used to calculate components of variations in unbalanced designs [19, 20]. The square root of variation was used for tables and plots to maintain interpretable values (severity grade).

**Table 1:** Grouping of telePRO outcomes by severity based on algorithm outcome colors in all questionnaires from each patient

Algorithm outcome color			Severity group
Green^a^ Severity grade 0	Yellow Severity grade 2	Red^b^ Severity grade 3	
1+	0	0	0
1+	1+	0	1
1+	0	1+	1.5
1+	1+	1+	1.7
0	1+	0	2
0	1+	1+	2.5
0	0	1+	3

Green, yellow, and red algorithm outcomes were assigned the severity grade values of 0, 2, and 3 to and reflect that the difference in consequences between a green and a yellow algorithm outcome is larger than the difference between yellow and red in all solutions. Each questionnaire can have one of three algorithm outcome colors, and hence patients with at least three answered questionnaires may have one of seven combinations of algorithm outcomes that define the patient’s severity group

^a^All items in algorithm with green color codes

^b^At least one item with red color code

#### Results

A total of 29 specific solutions in 24 diagnostic groups were included covering 42,015 referred patients from 89 hospital departments all over Denmark. One department may refer patients to more than one solution and the number of unique departments was 48 while the number of unique hospitals was 22. Also, the same patient may be referred to more than one solution, e.g., cancer patients may attend different solutions at different disease stages, one during active treatment and another during follow-up. Furthermore, patients may have several diseases corresponding to different solutions. There were 41,144 unique patients, 871 of whom had attended more than a single solution, and 16 had been referred to three solutions.

### Algorithm aims

The aims for the algorithms could be divided into four groups, shown by examples in Table 2 and tabulated for each solution in Table 3. The first aim, need of clinical attention (“Need”), represents the original purpose of AmbuFlex, namely PRO-based out-patient follow-up, where PRO, not hospital visits, form the basis for the contact. In some solutions, questionnaires with green algorithm outcome was handled automatically by AmbuFlex’ web-server, and a new questionnaire scheduled after a patient-specific assigned interval (e.g., 3 months) (*n* = 7 solutions, Table 2 and 3), while in 14 solutions questionnaires with green outcomes was reviewed and the green color used to support the decision if a visit was indicated or not. The second aim (“Path,” *n* = 3 solutions) used telePRO to select the most relevant type of clinical path, e.g., a telephone or in-clinic consultation with a doctor or a nurse. The third aim (“Treatment”, *n* = 2 solutions) used telePRO to decide if, e.g., planned antineoplastic treatment should be postponed. Frequently, side effects incompatible with a treatment are not discovered before the patient shows up for treatment, wasting time as well as expensive prepared medicine. The aim “Instruction” used algorithms to generate patient-specific on-screen messages or letters with instructions to the patient based on the PRO. This was implemented in three disease groups: bladder cancer [21, 22], immune therapy for malignant melanoma [23], and screening for depression in patients with ischemic heart diseases [24].

**Table 2:** Examples of aims of algorithm use in AmbuFlex telePRO solutions

Solution	Description and aim	Algorithm outcome		
ICD10 group (solution ID)		Green^a^	Yellow	Red^b^
Aim “Need”: need of clinical attention (automated cancellation if green algorithm outcome)				
C34 (PW) Lung cancer [25, 45]	Patients are referred after a CT scan showing non-progressive diseases and answer PRO once a week to detect progression	No contact. Automatic scheduling of next questionnaire	n/a	Possible disease progression. A clinician reviews PRO and EHR data and decides if need for earlier imaging
G40 (AE) Epilepsy [17, 34, 39, 43, 44, 46–48]	Patients answer PRO every 3, 6, 12, or 24 months. The purpose is to identify patients who need contact with the outpatient clinic	No contact. Automatic scheduling of next questionnaire	Possible need of contact. PRO and EHR data are reviewed. In 62% no further contact	Definite need of contact, e.g., episodes of seizures or planning of pregnancy
Aim “Need”: Need of clinical attention (decision support, all questionnaires are inspected by a clinician)				
E10 (DM) Type-1 DM [49, 50]	Patients answer PRO 2 weeks prior to preplanned consultation. The purpose is to identify and cancel unnecessary consultations	No obvious need of contact. 75% canceled without further contact	Possible need of contact. 49% canceled without further contact	Definite need of contact. No consultations were canceled
N18 (N2) Chronic kidney disease [26, 51]	Patients answer PRO every 3rd month. PRO and laboratory tests inform clinicians whether the patient needs contact with the outpatient clinic	No obvious need of contact. In 83%, no further contact before the next questionnaire	Possible need of contact and a clinician may call the patient. In 44%, no further contact	Definite need of contact. A clinician calls the patient or schedules a face-to-face consultation
Aim “Path”: Selection of relevant type of clinical contact (telephone/clinic or nurse/doctor)				
C50 (AB) Breast cancer	Patients attending follow-up answer PRO, which is used to evaluate need of consultation and indicate relevant clinician	Letter to the patient including medication for the next period	Telephone consultation with nurse	A doctor reviews PRO and EHR and decides further action
Aim “Treatment”: Treatment preparation				
C80 (IT/IN) Cancer NOS	Patients treated with immune therapy answer PRO 2 days before treatment. PRO is used together with blood test and EHR data for treatment adjustment	Treatment is ordered	Treatment is ordered. A clinician decides whether further action is necessary	Treatment not ordered. A clinician contacts the patient and decides further plan
Aim “Instruction”: Instructions or advice to the patient				
C67 (B3) Bladder cancer [21, 22]	Patients receiving chemo- or immunotherapy answer PRO with alert algorithm with on-screen pop-up instructions to the patient	No action	Advice to encourage self- treatment with supportive care advice	Advice to contact the department to obtain advice or hospitalized for the given treatment
I20 (AK) Ischemic heart disease [24]	Patients with atherosclerotic heart disease, cardiomyopathy, or heart failure answered PRO 4 weeks after discharge	Postal letter with test result. No further action	n/a	Postal letter to patient with test result and advice to contact family doctor

^a^All items with green color codes

^b^At least one item with red color code

*EHR* Electronic Health Record, *NOS* Not otherwise specified

**Table 3:** Characteristics of questionnaires and algorithms used in AmbuFlex telePRO solutions 2011–2021

Solution		Questionnaire		Algorithm			
ICD10 group (solution ID)	In operation	Content^a^	Items	Aim^b^	Items^c^	Patient override^d^	Color codes^e^
B20 HIV disease (HV)^g^	2015- >	[1, 3]	43	Path	32 items	M	GYR
C34 Lung cancer (PW)^f^	2018- >	EORTC [52] [2]	17	Need^auto^	12 items, f:1	M	GR
C43 Malignant melanoma (IM)^f^	2017–19		70	Instruction	24 items		GR
C50 Breast cancer (AB)	2016- >	EORTC CTCAE [53]	72	Need + Path	69 items	Q & M	GYR
C61 Prostate cancer (PC)	2014–19	EORTC [1]	73	Need	50 items, f:7	Q	GYR
C61 Prostate cancer (P2/P3)	2018- >	EORTC [2]	45	Need	38 items, f:2	Q & M	GYR
C67 Bladder cancer (B3)^f^	2019- > 21	CTCAE [2]	101	Instruction	37 items		GYR
C80 Cancer NOS (M3/KN)	2015- >		60	Treatment	57 items, f:2	M	GYR
C80 Cancer NOS (IT/IN)	2019- >		50	Treatment	47 items, f:1	M	GYR
E10 Type-1 DM (DM)^g^	2017- >	PAID [54] WHO5 [55] [1]	34	Need	28 items, f:2	Q & M	GYR
E66 Obesity (FF)	2021- >	[1]	18	Need	16 items, f:1	M	GYR
G35 Multiple sclerosis (SC)	2016- >	HAQ [56] WHO5 [1, 3]	52	Need	39 items, f:1	Q & M	GYR
G40 Epilepsy (AE/E3)^g^	2012- >	WHO5 [1, 3]	47	Need^auto^	38 items., f:2	Q & M	GYR
G40 Epilepsy (EP) (proxy)	2015- >		34	Need	27 items, f:1	Q &M	YR
G47 Sleep disorders (SN)	2013- >	ESS [57] [1, 3]	64	Need	49 items, f:3	Q & M	GYR
G47 Sleep disorders (SA)	2014- >	ESS [1, 3]	50	Need^auto^	34 items, f:1	Q & M	GYR
G47 Sleep disorders (NV)	2017- >	ESS, WHO5 [1, 3]	48	Need^auto^	36 items, f:2	Q & M	GYR
G91 Hydrocephalus (HC)	2017- >	WHO5	59	Need + Path	51 items, f:1	Q & M	GYR
I20 Ischemic heart ds. (AK)	2011–17	HADS [35]	14	Instruction	0 items, f:2		GR
J44 COPD (KO)	2015- >		13	Need^auto^	11 items, f:2	M	GYR
J45 Asthma (AT/A5)	2015- >	ACQ [58]	8	Need	8 items, f:3	Q & M	GYR
K50 Crohn’s disease (IB/I2)	2017- >	WHO5 [1, 3]	49	Need	46 items., f:5	Q & M	GYR
M05 Rheumatoid arthritis (RA/LG)^f^	2014- >	Flare [38]	40	Need	4 items, f:4	Q	GYR
M10 Gout (AU)	2020- >		30	Need	28 items, f:1	Q &M	GYR
M16 Arthrosis, hip (DP)^f^	2011–13	Oxford Hip [37] [1]	14	Need^auto^	0 items, f:1		GR
M17 Arthrosis, knee (DP)^f^	2011–13	Oxford Knee [36] [1]	14	Need^auto^	0 items, f:1		GR
N18 Chronic kidney ds. (N2)^g^	2018- >	EQ5D [59] [1, 3]	63	Need	27 items	Q	GYR
N80 Endometriosis (EN)	2020- >	WHO5 [1]	45	Need	35 items, f:1	Q & M	GYR
R52 Pain NOS (SM) (proxy)	2018- >		18	Need	10 items, f:9	M	GYR

General health items: [1] SF-36 GH1 [27], [2] EORTC QLQ C29 [52], [3] SF-36 HT [27]

^a^All solutions except ischemic heart disease (AK) included one or more additional single items in the algorithm

^b^Clinical purpose of algorithm. Need: need of clinical attention (auto: automatic cancellations if green algorithm outcome), Path: selection of relevant clinical path for contact, Treatment: treatment preparation, Instruction: instruction of the patient (cf. Table 2)

^c^Number of items included in the algorithm. *f* function depending on a combination of items

^d^The patient may override the algorithm by answering a specific question (Q) or enter any text into a text message field (M), which will induce both a red or yellow algorithm outcome

^e^Outcome colors used by the algorithm (cf. Fig 1)

^f^Research initiated

^g^Research enriched

### Diseases

TelePRO was implemented in a broad range of conditions including nearly all ICD-10 main groups, the highest number of solutions being in malignant (*n* = 8) and neurological diseases (*n* = 7) (Table 3). The most diverse use was in malignant diseases, which apart from out-patient follow-up also applied telePRO during active treatment (IT and M3, Tables 2 and 3) and to detect disease progression (PW). AmbuFlex is also used among cancer inpatients and patients attending palliative care, although without use of algorithms.

### Patients

The mean age of the referred patients was 57.2 years (SD 16.0 years) and 41.3% were women. The patient populations differed on nearly all parameters between the solutions (Table 4). The youngest patients were found in solutions for pain in children (SK) (10.6 years) and the oldest in chronic kidney disease (N2) (73.3 years). With respect to mortality of referred patients, the range was from 0 to 528 per 1000 patient years in patients with endometriosis (EN) and patients with lung cancer (PW), respectively. The patients submitted 171,268 questionnaires during a total observation time of 68,094 years. The longest follow-up time was in patients with epilepsy and sleep disorders, with a median follow-up of 4.3 and 4.0 years. The longest observation time (26,918 years, Table 4) was in sleep disorder (SA). The median number of questionnaires from each patient ranged from a single questionnaire to 86 in patients with COPD (KO). In lung cancer (PW), 55% of questionnaires came from patients delivering 50 or more responses (Table 5), while the same was the case for 96% in COPD (KO). At the beginning of the period, most responses were collected by paper questionnaires (up to 92% in the patients with knee arthrosis, a solution that ran from 2011 to 2013), while in the current solutions nearly all patients are contacted by secure e-mail and questionnaires are answered online. This significant development in our PRO data collection is described elsewhere [16].

**Table 4:** Characteristics of patients referred to AmbuFlex telePRO solutions 2011–2021

ICD10 group (solution ID)	Departments	Patients	Age (SD)	Gender	Mortality	Follow- up (yrs)	Observation
	*n*	*n*	Years	% Female	Per 1000 yrs	Median (max)	Years
B20 HIV disease (HV)	1	568	48.2 (12.3)	29.4	8	2.6 (5.9)	1082
C34 Lung cancer (PW)	8	230	67.2 (7.8)	60.0	528	0.4 (2.9)	154
C43 Malignant melanoma (IM)	1	72	62.4 (11.9)	52.8	158	0.4 (0.6)	23
C50 Breast cancer (AB)	1	1552	63.4 (11.7)	99.1	19	1.9 (4.9)	1801
C61 Prostate cancer (PC)	5	1273	65.1 (6.5)	0.0	7	0.7 (2.4)	838
C61 Prostate cancer (P2/P3)	5	2102	68.7 (8.0)	0.0	29	0.5 (3.0)	1089
C67 Bladder cancer (B3)	4	119	67.8 (9.0)	23.9	313	0.3 (0.7)	34
C80 Cancer NOS (M3/KN)	4	3917	63.1 (11.9)	62.3	131	0.4 (6.5)	3128
C80 Cancer NOS (IT/IN)	2	977	66.5 (10.6)	40.6	258	0.4 (2.0)	427
E10 Type-1 DM (DM)	1	290	47.1 (14.1)	47.6	5	2.7 (4.6)	706
E66 Obesity (FF)	1	60	43.2 (9.8)	76.7	0	0.1 (0.5)	5
G35 Multiple sclerosis (SC)	2	109	62.1 (9.0)	63.3	34	2.3 (3.7)	140
G40 Epilepsy (AE/E3)	4	6222	47.5 (18.9)	50.5	19	3.6 (9.8)	21,979
G40 Epilepsy (EP) (proxy)	3	231	43.5 (18.0)	44.2	31	2.5 (6.7)	508
G47 Sleep disorders (SN)	2	160	32.8 (11.9)	56.9	1	4.0 (7.8)	551
G47 Sleep disorders (SA)	5	12,188	56.3 (12.2)	26.3	7	2.9 (7.3)	26,917
G47 Sleep disorders (NV)	1	640	61.8 (11.5)	20.6	18	2.1 (4.4)	935
G91 Hydrocephalus (HC)	1	230	42.7 (18.4)	51.3	13	1.7 (4.2)	352
I20 Ischemic heart ds. (AK)	1	5000	66.2 (12.5)	40.6	38	0.0 (0.0)	0
J44 COPD (KO)	2	77	69.9 (8.5)	49.4	155	1.7 (6.3)	167
J45 Asthma (AT/A5)	4	228	48.8 (14.5)	61.4	0	0.9 (3.8)	245
K50 Crohn’s disease (IB/I2)	6	3203	46.3 (15.5)	55.5	2	1.5 (4.8)	4564
M05 Rheumatoid arthritis (RA/LG)	5	1178	62.5 (12.9)	69.8	14	1.5 (7.3)	2061
M10 Gout (AU)	1	72	60.2 (14.5)	13.9	19	0.2 (1.1)	15
M16 Arthrosis, hip (DP)	5	330	67.8 (10.9)	61.5	18	0.2 (1.1)	112
M17 Arthrosis, knee (DP)	5	475	67.3 (9.0)	57.7	15	0.2 (1.2)	152
N18 Chronic kidney ds. (N2)	3	45	73.3 (10.0)	33.3	29	1.2 (1.9)	54
N80 Endometriosis (EN)	1	116	35.9 (6.4)	100.0	0	1.0 (1.7)	46
R52 Pain NOS (SM) (proxy)	1	349	10.7 (3.1)	36.1	0	0.0 (2.2)	9

**Table 5:** TelePRO questionnaires processed in AmbuFlex telePRO solutions 2011–2021

ICD10 group (solution ID)	Questionnaires	Questionnaires per patient					Web^a^
	Total	Median	1	2–9	10–49	50+	
	*n*	*n*	%	%	%	%	%
B20 HIV disease (HV)	1370	2	12	88	0	0	100
C34 Lung cancer (PW)	8058	23	0	3	42	55	100
C43 Malignant melanoma (IM)	1193	17	0	8	92	0	100
C50 Breast cancer (AB)	3239	2	16	84	0	0	88
C61 Prostate cancer (PC)	2500	2	5	95	0	0	86
C61 Prostate cancer (P2/P3)	5050	2	10	90	0	0	93
C67 Bladder cancer (B3)	1515	11	1	23	76	0	100
C80 Cancer NOS (M3/KN)	26,546	6	1	44	39	16	100
C80 Cancer NOS (IT/IN)	6060	4	3	52	45	0	100
E10 Type-1 DM (DM)	2183	8	1	71	28	0	100
E66 Obesity (FF)	102	1	33	67	0	0	100
G35 Multiple sclerosis (SC)	230	2	19	81	0	0	72
G40 Epilepsy (AE/E3)	28,608	4	3	92	5	0	68
G40 Epilepsy (EP) (proxy)	708	3	8	92	0	0	39
G47 Sleep disorders (SN)	941	6	2	76	22	0	83
G47 Sleep disorders (SA)	36,309	3	10	90	0	0	85
G47 Sleep disorders (NV)	1532	2	14	86	0	0	89
G91 Hydrocephalus (HC)	859	3	5	92	3	0	100
I20 Ischemic heart ds. (AK)	5000	1	100	0	0	0	20
J44 COPD (KO)	14,249	86	0	0	4	96	100
J45 Asthma (AT/A5)	1121	3	3	66	31	0	100
K50 Crohn’s disease (IB/I2)	17,422	3	3	48	49	0	100
M05 Rheumatoid arthritis (RA/LG)	4136	2	6	73	21	0	89
M10 Gout (AU)	165	2	14	86	0	0	100
M16 Arthrosis, hip (DP)	332	1	100	0	0	0	16
M17 Arthrosis, knee (DP)	476	1	100	0	0	0	12
N18 Chronic kidney ds. (N2)	192	4	3	91	6	0	79
N80 Endometriosis (EN)	173	1	42	58	0	0	100
R52 Pain NOS (SM) (proxy)	999	2	10	89	1	0	100

^a^Percentage of internet-based responses

### The algorithms

The algorithms were unique for each solution because they are based on specific questionnaires [9, 17, 25, 26]. Examples of algorithms and meaning of color codes are shown in Table 2 and Supplemental Table 1. In four solutions, the core of the algorithm was based on group-validated questionnaires with fixed threshold values (Table 3). In the remaining solutions, no relevant instruments or threshold score values were available, and the algorithms were constructed as series of single items or scales, each addressing a clinical issue. We used SF-36 [27], SCL-90 [28] and the EORTC Item Library to select items [29]. If an item could not be located, a new item was created ad hoc, typically with response categories adapted from EORTC (“Not at all/A little/Quite a bit/Very much”). Questions regarding general health were collected from SF-36 [27]. At least one question regarding general health was asked in 19 (66%) of the solutions. All three colors were used in 23 solutions, green and red in 5, and yellow and red in one solution (Table 4).

### Algorithm outcomes

**Fig. 3:**
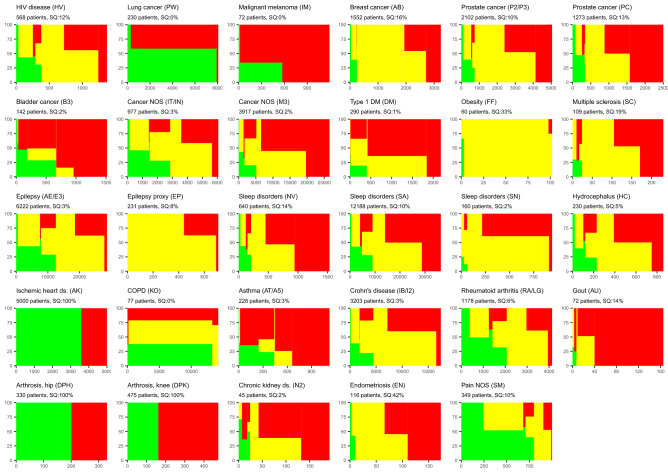
Distribution of PRO-algorithm outcomes. X-axis: number of questionnaires (algorithm outcomes), Y-axis: cumulative proportion of outcome colors. Prior to plotting, questionnaires were ordered patient-wise by outcome severity group (cf. Table 1), so that questionnaires from patients with least severe outcomes (solely green outcomes) appear on the left and questionnaires from patients with the most severe outcomes (solely red outcomes) on the right. SQ: Singleton questionnaires i.e., questionnaires from patients who so far have answered only one questionnaire and thus can possess no variation. (Color figure online)

**Fig. 4:**
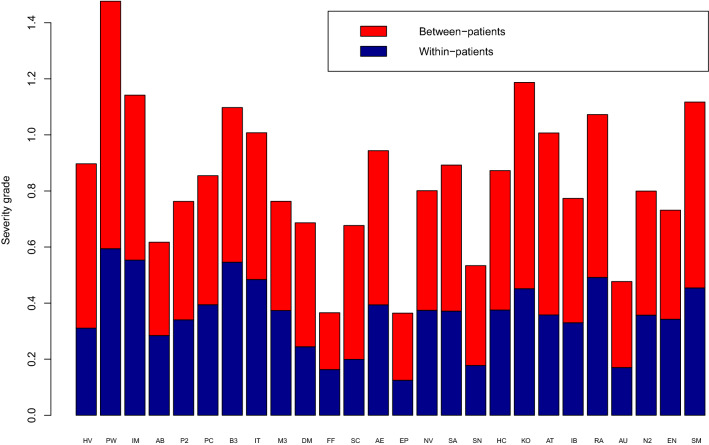
Standard deviation and components of variation (within- and between-patient) in algorithm outcome. Algorithm outcome for each questionnaire is measured as a discrete variable, severity grade, where green = 0, yellow = 2 and red = 3 (see Table 1). (Color figure online)

The algorithm outcomes for each solution are listed in Table 6. The content and purpose of the algorithms were heterogenic. Accordingly, the proportion of green outcomes varied between 1 and 59%. A graphical “fingerprint” of algorithm outcomes and intra-group variation is displayed in Fig. 3 for each solution. The total area of each color represents the proportion of that outcome. The within-group variation may be read vertically for each severity group. Some solutions were dominated by one algorithm outcome, e.g., breast cancer (AB) and ischemic heart disease (AK). No or little intra-patient variance (AK, DP) was seen if there was only a single questionnaire for each patient or the patient had been referred recently. In lung cancer (PW), more than 95% of the responses came from patients with variation in algorithm outcomes. Different solutions within the same patient group had similar “fingerprints” although questionnaires and algorithms differed (Table 3). In prostate cancer (P2/P3 and PC), the solutions had a similar distribution of outcomes and a similar pattern within severity groups. The most important difference was a larger proportion of patients with all-red algorithm outcomes in PC, which may reflect referral of more patients with advanced disease. The variation in outcomes (severity grade, defined in Table 1) is described in Table 6 and Fig. 4. The largest variation in severity was found in lung cancer (PW) and the lowest in the proxy solution in epilepsy (EP). After breaking down the total variation in within- and between-patient variation, the highest within-patient variation was 50% (bladder cancer, B3), while the lowest variation was 29% in patients with multiple sclerosis (SC).

**Table 6:** Variation in algorithm outcome in AmbuFlex telePRO solutions 2011–2021

ICD10 group (solution ID)	Algorithm outcome					Severity grade variation		
	Total	Green	Yellow	Red	Severity grade^a^	Total	Within-patient	Between-patient
	*n*	%	%	%	Mean		%	%
B20 HIV disease (HV)	1370	13	58	30	2.0	0.90	35	65
C34 Lung cancer (PW)	8058	59		41	1.2	1.48	40	60
C43 Malignant melanoma (IM)	1193	17		83	2.5	1.14	48	52
C50 Breast cancer (AB)	3239	4	69	27	2.2	0.62	46	54
C61 Prostate cancer (PC)	2500	8	39	53	2.4	0.85	46	54
C61 Prostate cancer (P2/P3)	5050	7	57	36	2.2	0.76	45	55
C67 Bladder cancer (B3)	1515	16	10	74	2.4	1.10	50	50
C80 Cancer NOS (M3)	26,546	6	36	58	2.5	0.76	49	51
C80 Cancer NOS (IT/IN)	6060	19	55	26	1.9	1.01	48	52
E10 Type 1 DM (DM)	2183	4	33	63	2.6	0.69	36	64
E66 Obesity (FF)	102	2	97	1	2.0	0.37	45	55
G35 Multiple sclerosis (SC)	230	3	54	43	2.4	0.68	29	71
G40 Epilepsy (AE/E3)	28,608	18	63	20	1.8	0.94	42	58
G40 Epilepsy proxy (EP)	708		84	16	2.2	0.36	34	66
G47 Sleep disorders (SN)	941	1	66	33	2.3	0.53	33	67
G47 Sleep disorders (SA)	36,309	10	44	46	2.3	0.89	42	58
G47 Sleep disorders (NV)	1532	6	36	58	2.5	0.80	47	53
G91 Hydrocephalus (HC)	859	10	51	39	2.2	0.87	43	57
J44 COPD (KO)	14,249	35	43	22	1.5	1.19	38	62
J45 Asthma (AT/A5)	1121	12	10	78	2.5	1.01	36	64
K50 Crohn’s disease (IB/I2)	17,422	8	62	30	2.1	0.77	43	57
M05 Rheumatoid arhritis (RA/LG)	4136	27	54	19	1.6	1.07	46	54
M10 Gout (AU)	165	1	12	87	2.8	0.48	36	64
N18 Chronic kidney ds. (N2)	192	6	29	65	2.5	0.80	45	55
N80 Endometriosis (EN)	173	3	46	50	2.4	0.73	47	53
R52 Pain NOS (SM) (proxy)	999	52	39	8	1.0	1.12	41	59

Solutions with only one questionnaire per patient are not included

^a^See Table 1

## Discussion

TelePRO has been applied in 29 specific solutions of AmbuFlex in 24 different patient groups, thus covering 12 of the first 19 ICD chapters. There were large variations between solutions with respect to patient characteristics (ICD10 group, age, gender, mortality) as well as questionnaire- and algorithm content and algorithm outcomes.

### Variation in algorithm outcomes

Variations in algorithm outcomes may be divided into within-patient, between-patient and between-solution. Except for screening purposes with just one measurement, a certain degree of within-patient variation over time is a prerequisite in repeated measurements and was met in most solutions while the considerable between-patient and between-solution merely is a marker for the wide range of applicability of algorithm-based telePRO.

### The four different aims of telePRO

Aim “Need”, where telePRO is used to evaluate the patient’s need for clinical attention, was used in the majority of the implementations. Denis et al. evaluated weekly symptoms reported by patients with lung cancer [12]. Twelve symptom items automatically triggered an alert to the clinicians if a pre-defined threshold was exceeded. A similar set-up was described in a study by Basch et al. [13]. In this study, patients could weekly self-report side effect symptoms after chemotherapy, and e-mail alerts were sent to clinicians if symptom scores worsened by a pre-defined threshold. Armstrong et al. described use of remote PRO with a mobile app during the first 30 days following ambulatory breast reconstruction [11]. Patients reported pain on a visual analog scale and quality of recovery on a nine item questionnaire daily for 2 weeks and thereafter weekly for 2 weeks. Clinicians were alerted by red flags, and abnormally high pain scores or low recovery scores prompted in-person follow-up. A similar approach was applied in an Australian study [30]. Brundage et al. summarize experiences [31] and point out that if PRO data are used remotely between visits, it is important to use pre-defined threshold levels. Decisions regarding the definition of these thresholds must be made by experts with sufficient expertise to weigh the implications of false-positive versus false-negative alerts [32]. In AmbuFlex, clinical experts are involved in defining the PRO-based algorithm thresholds and decide whether a specific response category should be given a green, yellow, or red color. In solutions where green outcomes are handled automatically (“Need^auto^”), the risk of false negative cases is more important than false-positive cases and a high sensitivity should be a key consideration. Regarding the aim “Instruction,” the telePRO algorithm generates an instruction to the patient instead of an alert to the clinician, which basically poses the same demands of sensitivity. PRO-based alerts in the “eRAPID” system [33] included PRO data about adverse events related to chemotherapy treatment. The system provided tailored feedback to patients if they reported severe symptoms. In the case of less severe symptoms, the patients were asked to follow self-management advice. Thus, alerts based on PRO data can be tailored not only to clinicians but also to patients. As pointed out by Brundage et al., considerations regarding defining clinical alerts and threshold levels should be based on when, how, and to whom alerts are directed and whether PRO data are combined in the algorithm with other important data, e.g., a blood test or data from the patients' medical record [31]. In the two aim types (“Path” and “Treatment”), all questionnaires are individually evaluated and therefore false negatives are less problematic.

### Limitations

Out-patient groups are the main target for telePRO-based follow-up, but not all diseases and patients are suitable. For a disease to be relevant, evaluation of the patient’s state must rely on measures reportable as PRO, which may also include self-measurements. In two solutions we were able to identify the source population of referred patients; rheumatoid arthritis (RA) and epilepsy (AE). Successful referral was related to young age and low disease activity [9, 34] and higher socioeconomic status [34]. Target groups was not intended to include very sick patients and a solution should not be a “one-size-fits-all”. Each patient should be evaluated before referral and allowed to return to standard follow-up whenever he or she wishes to do so. This is for ethical reasons, but is also a way to monitor and evaluate the telePRO solution. PRO-based follow-up requires a mentally capable patient. However, in patient groups with mentally disabled persons, proxy versions of the questionnaire may be applied. We did this in the pain in children (SM) and patients with epilepsy (EP) solutions in 231 referred patients compared to 6222 in the main solution (AE) [17].

### Questionnaires and algorithms

Traditionally, validated questionnaires are validated for purposes other than telePRO, where the main question in aim “Need” may be expressed as: “Does this patient need clinical attention at the moment?”, in aim “Path”: “Which type of clinical contact is most relevant?”, in aim “Treatment”: “Is this patient ready for the planned treatment?”, and in aim “Instruction”: “What is the most relevant instruction to the patient?”. We based the algorithm on a traditionally validated questionnaire and cut-off values in screening for depression [35], hip and knee alloplastic operations [36, 37], and rheumatoid arthritis [38]. In all other solutions, algorithms were based on series of single items adapted from item libraries or developed together with clinicians [39]. When using the single-item approach, each item is provided its own cut-off value, making it possible for clinicians to achieve consensus regarding items, cut-off values, and hence the whole algorithm. This process runs in parallel with the development and revision of the questionnaire and takes years to maturate. The first epilepsy solution (AE) was launched in 2011 and has been revised four times. After 5 years without any changes, a national revision is now in progress.

### Length of questionnaires

Doctors and nurses will often focus on the length of the questionnaire as a critical factor and on the clinical relevance of each item. From our experience, patients are more concerned with the last issue than the first and patients accept long questionnaires if they find the questions relevant. Questionnaires in research-initiated solutions are often longer, which may be accepted by the participating patient because they volunteered to participate, while several of the clinical solutions have become standard care and the patient has to explicitly opt out. A good reason for clinicians to prefer short questionnaires is that both patient and clinicians will expect action to be taken if the patient reports a problem. Examples are depressive symptoms or sexual problems in solutions in specialized departments, where some clinicians expecting such issues to be handled by the family doctor. There is no simple solution to this problem. In some cases, explicit guidelines have been developed [40, 41].

### TelePRO vs PRO for consultation support

In most AmbuFlex telePRO solutions, PRO is also used as a tool to enhance the consultation process. During the last decade, an increase in the use of PRO at the patient level has been seen in clinical care. However, PRO has no value in itself; it is the context and actual use that makes the difference. If PRO is an add-on to existing clinical practice, the implementation is very dependent on the commitment of the individual clinicians and in some implementations only a minor part of responses are ever seen by a clinician [16, 42]. In telePRO-based follow-up, PRO constitutes the basis itself for the follow-up. Each time a questionnaire is received, it is either handled automatically (green response) or put on an alert list, like incoming lab tests, where it remains until a clinician has reviewed it and decided whether the patient should be contacted or not. Therefore, virtually all questionnaires are used: automatically, as a decision tool, and/or as a basis for patient-clinician interaction in the consultation.

### Patient safety

Questionnaires with a calculation of scores or a color code for decision aid are considered medical devices if collected electronically and used in the treatment of patients. As such, telePRO solutions must ensure patient safety and be compliant with EU legislation for Medical Device Regulatory (MDR). Patient safety is a cornerstone, also within the application of PRO in clinical practice. The questionnaire and color code must uncover the defined aim and be understandable and meaningful to patients and clinicians, and the IT system must be reliable and secured. There are standards for the development and test of IT systems, while it is an ongoing process to decide how to validate questionnaires and algorithms, especially with respect to the green algorithm outcomes, where the patient may not be contacted. We are in the middle of this process. In outpatient follow-up, patients are instructed to contact the department, emergency room, or their family doctor in event of sudden health deterioration between appointments. This also solves a potential hazard for PRO-based follow-up if a questionnaire is lost for some reason. In most solutions, non-responding patients are appointed a specific code on the alert list. Also, only patients capable of evaluating and reporting their health should be referred.

### The patient perspective

Two of the aims of AmbuFlex are to optimize the use of resources and to promote patient-centered care. Is there a contradiction between the patient’s interests and the interests of clinicians and hospital owners? In AmbuFlex’s very first years, health administrators and hospital owners in Denmark to some degree considered AmbuFlex as an easy way to cancel appointments for patients with no or little need of clinical attention, but did not acknowledge the resources needed to implement and run it. This view has changed, and telePRO is now merely seen as a tool for achieving better quality of care. Few patients are interested in fixed consultations when there is no need [43] and such patients should be offered standard follow-up. Clinicians also need to see less complicated cases to be able to experience the whole spectrum of a disease; otherwise, they will develop a biased picture of prognosis [44].

## Conclusion

TelePRO can be applied in any setting where PRO can be used to evaluate patient clinical status and needs. Solutions are unique with respect to questionnaire content, algorithms, clinical purpose, and patient characteristics. Questionnaires and algorithms should be adapted for each specific patient group and aim.

## Supplementary Information

Below is the link to the electronic supplementary material.

Supplementary file1 (DOCX 22 kb)

## Data Availability

The data that support the findings of this study are available on request from the corresponding author [NHH]. The data are not publicly available due to restrictions e.g. their containing information that could compromise the privacy of participants.
