# Supplementary material for: Patient-reported outcome (PRO) measurements in chronic and malignant diseases: ten years’ experience with PRO-algorithm-based patient-clinician interaction (telePRO) in AmbuFlex
Source: Qual Life Res. 2023 Jan 13;32(4):1053–67. doi: 10.1007/s11136-022-03322-9 (PMC10063508; doi:10.1007/s11136-022-03322-9)
Supplement: Supplementary file 1 — Supplementary file1 (DOCX 22 kb) [file 11136_2022_3322_MOESM1_ESM.docx]

**Supplemental Table 1. Items in algorithms and color codes in three selected AmbuFlex telePRO solutions.**

**Epilepsy (AE/A3)**

| **Item** | **Color codes** | | | | |
| --- | --- | --- | --- | --- | --- |
| Number of seizures during the last year | 0 | > 0 |  |  |  |
| Number of absence seizures during the last 3 months | 0 | > 0 |  |  |  |
| Number of generalized seizures during the last 3 months | 0 | > 0 |  |  |  |
| Seizure impairment | Yes | No |  |  |  |
| Seizure injury | No | Yes, but not serious | Serious damage |  |  |
| Emergency room visit due to epilepsy | Yes | No |  |  |  |
| Relatives' worried | Never | Rarely | Occasionally | Frequently | Don't know |
| Headaches | Never | Occasionally | Sometimes | Often | Very often |
| Dizziness | Never | Occasionally | Sometimes | Often | Very often |
| Tremor/shaking | Never | Occasionally | Sometimes | Often | Very often |
| Double vision | Never | Occasionally | Sometimes | Often | Very often |
| Loss of appetite | Never | Occasionally | Sometimes | Often | Very often |
| Eating too much | Never | Occasionally | Sometimes | Often | Very often |
| Difficulty remembering | Never | Occasionally | Sometimes | Often | Very often |
| Difficulty concentrating | Never | Occasionally | Sometimes | Often | Very often |
| Aggression | Never | Occasionally | Sometimes | Often | Very often |
| Fatigue | Never | Occasionally | Sometimes | Often | Very often |
| Sadness | Never | Occasionally | Sometimes | Often | Very often |
| Fear of having seizures | Never | Occasionally | Sometimes | Often | Very often |
| Problems with sexuality | Never | Occasionally | Sometimes | Often | Very often |
| Being suicidal | Never | Occasionally | Sometimes | Often | Very often |
| Well-being WHO-5 Index | Score < 50 or just one extreme answer | | | | |
| General health | Excellent | Very good | Good | Fair | Poor |
| General health compared to last year | Much better | Somewhat better | About the same | Somewhat worse | Much worse |
| Medication adherence | Daily | Weekly | Monthly | Never / very rarely | |
| Side effects | No | Yes, a few | Yes, some | Yes, many |  |
| Work less because of epilepsy | Yes | Partly | No |  |  |
| Social limitations | No | Yes |  |  |  |
| Alcohol consumption | > consume 14/21 units a week | | | | |
| Use of recreational drugs | Never | Monthly | Weekly | Daily |  |
| Pregnant | Yes | No |  |  |  |
| Planning pregnancy | Yes | No |  |  |  |
| Car driving last month | Yes (+ seizures) | No |  |  |  |
| Message to the clinic (free text) | Any topic | None |  |  |  |
| What is your current need of contact with the outpatient clinic? | I will call the clinic if I need a contact | I would like a clinician to call me | I wish for an appointment in the clinic | I don´t know |  |

**Lung cancer (PW)**

| **Item** | **Color codes** | | | | | | | | | | | | | | |
| --- | --- | --- | --- | --- | --- | --- | --- | --- | --- | --- | --- | --- | --- | --- | --- |
|  | Very poor | | | | |  | | | Excellent | | | | | | |
| How would you rate your overall health during the past week? | 1 | 2 | | | 3 | | 4 | | | 5 | | | 6 | | 7 |
|  | Not at all | | A little | Quite a bit | | | | Very Much | | | |  | |  | |
| Were you short of breath? | 1 | 2 | | | 3 | | 4 | | |  | | |  | |  |
| Have you had pain? | 1 | 2 | | | 3 | | 4 | | |  | | |  | |  |
| Were you tired? | 1 | 2 | | | 3 | | 4 | | |  | | |  | |  |
| Have you lacked appetite? | 1 | 2 | | | 3 | | 4 | | |  | | |  | |  |
| How much did you cough? | 1 | 2 | | | 3 | | 4 | | |  | | |  | |  |
| Did you cough up blood? | 1 | 2 | | | 3 | | 4 | | |  | | |  | |  |
| Body temperature | ≥38.2 |  | | |  | |  | | |  | | |  | |  |
| Hoarse voice worsened during the past week? | Yes |  | | |  | |  | | |  | | |  | |  |
| Facial swelling worsened during the past week? | Yes |  | | |  | |  | | |  | | |  | |  |
| Do you sense a growing tumor? | Yes |  | | |  | |  | | |  | | |  | |  |
| How much do you weigh? | ≥ 3 kg weight loss compared to first measure | | | | | | | | | |  | |  | | |
| In the past week, have you had other symptoms that you think may be associated with your cancer? (Free text) | | | | | | | | | | | Any text | |  | | |

**Chronic kidney disease (N2)**

| **Item** | **Color codes** | | | | | |
| --- | --- | --- | --- | --- | --- | --- |
| Lack of appetite | Not at all | Somewhat | Moderately | Very much | Extremely |  |
| Aversion to food | Not at all | Somewhat | Moderately | Very much | Extremely |  |
| Feeling of unease | Not at all | Somewhat | Moderately | Very much | Extremely |  |
| Nausea | Not at all | Somewhat | Moderately | Very much | Extremely |  |
| Vomiting | Not at all | Somewhat | Moderately | Very much | Extremely |  |
| Itchy skin | Not at all | Somewhat | Moderately | Very much | Extremely |  |
| Shortness of breath | Not at all | Somewhat | Moderately | Very much | Extremely |  |
| Swollen legs | Not at all | Somewhat | Moderately | Very much | Extremely |  |
| Dizziness | Not at all | Somewhat | Moderately | Very much | Extremely |  |
| Difficulty remembering | Not at all | Somewhat | Moderately | Very much | Extremely |  |
| Difficulty concentrating | Not at all | Somewhat | Moderately | Very much | Extremely |  |
| Restless legs discomfort? | All of the time | Most of the time | A good bit of the time | Some of the time | A little of the time | None of the time |
| How much of the time did you feel tired? | All of the time | Most of the time | A good bit of the time | Some of the time | A little of the time | None of the time |
| How much physical pain have you had? | None | Very mild | Mild | Moderate | Severe | Very severe |
| Were you limited in doing regular daily activities? | Not at all | A little | Quite a bit | Very much |  |  |
| Have you been constipated? | Not at all | A little | Quite a bit | Very much |  |  |
| Have you had diarrhea? | Not at all | A little | Quite a bit | Very much |  |  |
| Have you had to urinate frequently at night? | Not at all | A little | Quite a bit | Very much |  |  |
| Have you had trouble sleeping at night? | Not at all | A little | Quite a bit | Very much |  |  |
| Were you worried about your future health? | Not at all | A little | Quite a bit | Very much |  |  |
| How often do you have difficulty remembering to take all your medication? | Daily | Weekly | Monthly | Never/Rarely |  |  |
| In general, would you say your health is: | Excellent | Very good | Good | Fair | Poor |  |
| Compared to 1 year ago, how would you rate your health is general now? | Much better | Somewhat better | About the same | Somewhat worse | Much worse |  |
| Do you experience other symptoms? | No | Yes, [Free text] |  |  |  |  |
| Message to the clinic (free text) | Any text |  |  |  |  |  |
| What is your current need of contact with the outpatient clinic? | I will call the clinic if I need a contact | I would like a clinician to call me | I wish for an appointment in the clinic | I don´t know |  |  |

Green No need of contact with the outpatient clinic with respect to this item.

Yellow May need contact with respect to this item.

Red: Need of contact with the clinic with respect to this item.

Note: For information regarding time reference and exact wording of questions and response categories, consult the individual papers.
